# Impact of COVID-19 on Pediatric Primary Care Visits at Four Academic Institutions in the Carolinas

**DOI:** 10.3390/ijerph18115734

**Published:** 2021-05-27

**Authors:** Callie L. Brown, Kimberly Montez, Jane Blakely Amati, Kristina Simeonsson, John D. Townsend, Colin J. Orr, Deepak Palakshappa

**Affiliations:** 1 Department of Pediatrics, Wake Forest School of Medicine, Winston-Salem, NC 27101, USA; kmontez@wakehealth.edu; 2 Department of Epidemiology and Prevention, Wake Forest School of Medicine, Winston-Salem, NC 27101, USA; dpalaksh@wakehealth.edu; 3 Department of Pediatrics, Prisma Health, University of South Carolina School of Medicine Greenville, Greenville, SC 29605, USA; Blakely.Amati@prismahealth.org; 4 Department of Pediatrics, East Carolina University, Greenville, NC 27834, USA; SIMEONSSONK@ecu.edu (K.S.); TOWNSENDJ16@ECU.EDU (J.D.T.); 5 Department of Pediatrics, University of North Carolina at Chapel Hill, Chapel Hill, NC 27514, USA; cjo1@email.unc.edu; 6 Department of Internal Medicine, Wake Forest School of Medicine, Winston-Salem, NC 27101, USA

**Keywords:** COVID-19, pediatrics, primary care, well visits, mental health, obesity

## Abstract

We aimed to determine how COVID-19 affected the number and type of pediatric primary care visits in April 2020, compared to April 2019, and which characteristics were associated with obtaining care in 2020. We performed a retrospective chart review of patients receiving care in April 2019 and April 2020 from four large, academic institutions across two states. The subjects were included if they were aged 0–18 years and were seen in a pediatric clinic in April 2019 or April 2020. We extracted the number of visits, visit type, and visit diagnosis; and the patient characteristics, including age, race/ethnicity, and insurance status. Logistic regression analysis identified characteristics associated with obtaining care in April 2020. We included 120,230 visits. Participants were 50% white and half had Medicaid. In 2020 there were significantly fewer visits for both well and acute visits with 42,670 visits in 2020 compared to 77,560 in 2019; 6616 were telehealth visits in 2020. Visits for chronic conditions were significantly decreased in 2020. Attending a visit in 2020 was more likely if the participant was black or Hispanic, younger, attending an acute visit, or had private insurance. During the COVID-19 pandemic, pediatric primary care decreased substantially for both well visits and follow-up of chronic conditions.

## 1. Introduction

On 13 March 2020, the World Health Organization declared the SARS-coronavirus-2 (SARS-CoV-2) disease 2019 (COVID-19) a global pandemic. The first case of COVID-19 was reported in the United States (U.S.) on 20 January 2020, and the U.S. declared a national emergency on 13 March 2020. As of 19 April 2021, there were over 31 million confirmed cases and over 560,000 deaths due to COVID-19 in the U.S. [1]. Fortunately, infection with SARS-CoV-2 causes less severe disease in children and adolescents compared to adults. Due to the social and economic impact of COVID-19, however, the pandemic has led to numerous indirect effects in children and adolescents, including missed days of school, worse diet-related behaviors, and increasing rates of anxiety and depression [2,3,4]. Furthermore, recent research indicates these impacts have disproportionately affected racial/ethnic minorities in the U.S. [5,6,7]. 

In the U.S., children are typically seen for routine primary care by a general pediatrician or family physician. Well child visits are performed annually for children ages 2–18 and every 2–6 months during infancy. The COVID-19 pandemic has significantly impacted healthcare utilization among children. The pandemic has resulted in a significant decrease in pediatric emergency department visits and hospitalizations. [8,9,10,11]. There has also been a decrease in pediatric primary care visits due to concerns about children becoming infected with SARS-CoV-2, or in order to comply with stay-at-home orders [12]. During March and April 2020, restaurants, bars, public schools, and nonessential businesses were closed for in-person services and large gatherings were prohibited. Although health systems remained open during this time, most nonurgent visits, including primary care visits, were cancelled or rescheduled.

These missed primary care appointments have led to missed immunizations, delayed presentation of new onset medical conditions (e.g., Type 1 diabetes), and worse chronic disease management (for conditions such as obesity, ADHD) [13,14,15,16]. Telehealth has emerged as a tool for patients to access primary care during the pandemic, although how much impact telehealth has had on closing the gap in pediatric primary care is still unclear. While there has been growing literature about the impact of the COVID-19 pandemic on pediatric healthcare utilization [8,12,17,18,19,20], there are still limited data on the effect of the pandemic on the volume of pediatric primary care visits and the characteristics of patients who did and did not attend a visit during the pandemic. 

To fill this gap in the literature, the objective of this study was to determine the association of pediatric primary care delivery and the COVID-19 pandemic. Using data across four large academic medical centers that include data from 79 clinics serving North and South Carolina, we specifically evaluated the number of children who missed primary care visits and which visit types and diagnoses were most affected. Additionally, we evaluated whether attending visits during the pandemic was associated with certain patient characteristics and the effect of telehealth on closing the gap in pediatric primary care visits.

## 2. Methods

We retrospectively extracted electronic health record (EHR) data from Wake Forest Baptist Health/Wake Forest School of Medicine, University of North Carolina at Chapel Hill Health, East Carolina University Clinics, and Prisma Health/University of South Carolina School of Medicine Greenville. These institutions represent three of the four large academic medical centers in North Carolina, and Prisma Health is one of the two large academic medical centers in South Carolina. Subjects were included if they were aged 0–18 years and were seen in a pediatric clinic at one of these medical centers in April 2019 or April 2020. We extracted data from 79 primary care clinics that were members of these medical centers. Extracted elements included the number and type of primary care visits (categorized as well, acute, video, or phone visits); patient characteristics, including age, race, ethnicity, and insurance status; and visit diagnosis. The type of primary care visit was categorized as “well” if well visit was listed as a visit type or as a visit diagnosis. Video and phone visits were not further designated as well or acute visits as this was not done uniformly across institutions. Race/ethnicity were combined into non-Hispanic white, non-Hispanic black, Hispanic, and other categories and insurance were categorized as Medicaid, other public, private, or none. 

We used descriptive statistics to describe the sample. Group comparisons by year were performed using chi square tests or t-tests, for categorical or continuous variables, respectively. Logistic regression analysis identified the characteristics associated with the odds of obtaining care in April 2020. We also used separate logistic regression models to examine the characteristics associated with visit attendance of specific visit types in 2020. Characteristics included in the multivariate analyses were sex, race/ethnicity, insurance status, age, visit type, and site. Data analysis was performed using Stata SE v14.2 (StataCorp LLC (College Station, TX, USA)). 

## 3. Results

### 3.1. Patient Characteristics

Patients with data extracted from the EHR (*n* = 73,465) were 50% male, 44% non-Hispanic white, 20% non-Hispanic black, and 17% Hispanic and had a mean age of 7.2 years (SD 5.7). About half of children (54%) were insured by Medicaid. Patient characteristics by visit year are shown in Table 1. There were 52,339 unique patients seen in 2019 compared to only 21,126 seen in 2020. Compared to 2019, patients attending visits in 2020 had a similar distribution by sex. There was a larger percentage of patients attending visits in 2020 who were non-Hispanic black, Hispanic, or of unknown race and who had other public or no insurance. Patients attending visits in 2020 were younger than those in 2019.

### 3.2. Visit Characteristics by Year

There were 120,230 total visit encounters: 15% well visits, 79% acute visits, 2% phone visits, and 4% video visits. Encounter characteristics by visit year are shown in Table 2. There were 77,560 visit encounters in 2019 and only 42,670 in 2020. Compared to 2019, there was a smaller percentage of well and acute visits in 2020 but 2020 saw the introduction of both phone and video visits, although these visit types accounted for only 2199 and 4417 visits, respectively. There were significantly fewer visits in 2020 for well visits, asthma, obesity, attention deficit hyperactivity disorder, depression, and anxiety.

### 3.3. Characteristics Associated with Visit Attendance in 2020

Patients also had increased odds of attending a visit in 2020 compared to in 2019 if they were non-Hispanic black, Hispanic, or of unknown race/ethnicity (compared to non-Hispanic whites) and had other public, private, or no insurance (compared to Medicaid) (Table 3). Patients also had decreased odds of attending visits with increasing patient age. There were lower odds of attending well visits compared to acute visits in 2020. 

### 3.4. Characteristics Associated with Visit Attendance of Specific Visit Types in 2020

The odds of patients attending a visit with a specific visit diagnoses are shown in Table 4. Males had lower odds of attending well visits and visits for depression but had higher odds of attending visits for asthma and ADHD. Patients with a non-Hispanic black race/ethnicity had higher odds than those with a non-Hispanic white race/ethnicity of attending well visits and asthma visits and Hispanic patients had higher odds of attending a visit for obesity. Both non-Hispanic black and Hispanic patients had lower odds than children of white race/ethnicity of attending visits for ADHD, depression, and anxiety. Compared to children insured by Medicaid, children insured by private insurance had higher odds of attending well visits and visits for depression and anxiety and lower odds of attending visits for asthma, obesity, ADHD, and depression. Children were less likely to attend a telehealth visit (phone or video visit) if the child was female, had a non-Hispanic black or other race/ethnicity, were younger, or had Medicaid or no insurance.

## 4. Discussion

During the COVID-19 pandemic, pediatric primary care at four U.S. academic institutions decreased substantially for both well visits and follow-up visits for chronic conditions. Telehealth visits, including phone and video visits, were newly utilized in 2020. Compared to non-Hispanic white patients, racial/ethnic minority populations had higher odds of attending visits in 2020.

Our study is consistent with prior literature reporting a decrease in healthcare utilization during the pandemic, particularly hospitalizations and emergency department visits. [8,9,10,11]. However, our study adds to the sparse literature, by evaluating 79 clinics across four large, academic institutions in the Southeastern US, indicating decreased utilization of pediatric primary care [12,19]. Reasons for decreased primary care visits include patients’ fear of contracting the SARS-CoV-2 infection [11,18], practices deferring elective visits [21], governmental stay at home orders [9], and decreased infection-related visits [22]. These missed primary care appointments have the potential to lead to long-lasting detrimental health effects in children. Missed primary care visits contribute to gaps in childhood immunizations [22], which may lead to an increase in vaccine-preventable infections [23]. Decreased visits for diseases such as obesity and asthma may lead to worse chronic disease management, as reflected in worsening obesity rates throughout the pandemic and will be very challenging to reverse [24]. Additionally, fewer provider visits may contribute to delays in the diagnosis of new onset health conditions; for example, patients with a new diagnosis of Type I diabetes mellitus were more likely to present in severe diabetic ketoacidosis in 2020, compared to 2019 [25]. 

Our study demonstrates the emergence of telehealth as a healthcare delivery tool to increase patients’ access to primary care. Medicaid and private insurers expanded coverage and relaxed requirements about telehealth use during the pandemic, and health organizations like the American Academy of Pediatrics promoted its use, triggering many practices to develop telehealth programs [26]. Our study mirrors others reporting unprecedented expansion of telehealth visits [12,13,20,27]. While clinics across the country made tremendous efforts to safely contact patients via telehealth in accordance with national governmental recommendations and local governmental restrictions, the telehealth visits did not come close to bridging the gap between pre- and post-pandemic clinic numbers. Additional longitudinal research is necessary to examine how telehealth has expanded over the course of the pandemic and its potential to increase patient access. Telehealth has the opportunity to not only improve healthcare access, but population health, chronic disease management, and medication adherence, not to mention supporting value-based care [28]. Furthermore, several studies have demonstrated pediatricians’ acceptance of telehealth during the pandemic [27,29]. Continued reimbursement after the pandemic ends is essential for expanded access and utilization and is an opportunity for advocacy. In the instances of future pandemics, telehealth use could be expanded to include well child visits, ensuring that appropriate screenings for developmental delays are not missed. Health systems should also consider the safety and accessibility of their clinic spaces (e.g., separating well from sick families in the waiting room, administration of vaccines and testing in clinic parking lots or cars, etc.) and having a larger back stock of personal protective equipment available for providers. It is imperative that families feel safe coming to see their pediatric provider to ensure that important preventative health care is not missed during these times.

In our study, racial/ethnic minority populations had higher odds of attending any visit in 2020 compared to non-Hispanic white patients. Prior literature has shown that racial/ethnic minorities experienced smaller declines of in-person visits during the pandemic [30]. Possible reasons for increased odds of in-person visits in 2020 among racial/ethnic minorities include less fear of attending in-person visits, disparities in knowledge regarding the pandemic [31], differences in access to telehealth, or practice-level differences in patient scheduling, such as encouraging in-person visits for certain patients. When examining specific visit types, however, racial/ethnic minority patients had significantly lower odds of attending in-person mental health-related visits compared to non-Hispanic whites, a known existing disparity in the literature prior to the pandemic, possibly due to stigma [6]. Lastly, compared to non-Hispanic white patients, black and other patients had significantly lower odds of attending a telehealth visit, which has been previously reported [32], possibly due to inequities in broadband access, available technology, mistrust [33,34], or provider biases.

Our study has several limitations that should be noted. First, the study was conducted in the Southeastern U.S. with a primarily Medicaid-insured population, so results may not be generalizable nationally or internationally. Second, we only compared one month from each year rather than longitudinal data, so we are unable to determine the impact of the COVID-19 pandemic on healthcare utilization throughout 2020 and 2021. Third, we only examined the healthcare system-level data, so we are unable to determine the impact on individual health outcomes, such as missed vaccines, delayed referrals and severity of disease, but our study does provide important data regarding impact on primary care utilization. Fourth, data extraction was conducted by each individual institution, and although the data requested were specific, this may have resulted in incomplete data.

## 5. Conclusions

In conclusion, pediatric primary care utilization at four U.S. academic institutions in the Southeastern U.S. decreased substantially for both well and follow-up visits. Future studies should evaluate whether these visits were made up later in 2020, either in person or via telehealth, or missed entirely and explore what health consequences the children experienced (e.g., missed vaccinations, delayed referral for developmental delays, or increased overweight/obesity) due to this missed routine care.

## Figures and Tables

**Table 1:** Participant characteristics by year.

Characteristic	April 2019 *n* = 52,339 (71.2%) *n* (%) or Mean (SD)	April 2020 *n* = 21,126 (28.8%) *n* (%) or Mean (SD)
Site (*n* = 73,465)		
1	15,193 (29.0%)	5076 (24.03%)
2	3136 (6.0%)	1319 (6.2%)
3	1978 (3.8%)	795 (3.8%)
4	32,032 (61.2%)	13,936 (66.0%)
Male sex (*n* = 73,193)	26,639 (50.9%)	10,801 (51.1%)
Race/ethnicity (*n* = 73,201)		
Non-Hispanic white	23,741 (45.4%)	8.922 (42.2%)
Non-Hispanic black	10,177 (19.4%)	4246 (20.1%)
Hispanic	9005 (17.2%)	3697 (17.5%)
Other	5313 (10.1%)	2047 (9.7%)
Unknown	4103 (7.8%)	2214 (10.5%)
Insurance (*n* = 73,181)		
Medicaid	28,311 (54.1%)	11,090 (52.5%)
Other public	2458 (4.7%)	1020 (4.8%)
Private	19.914 (38.1%)	7695 (36%)
None	1632 (3.1%)	1322 (6.3%)
Age in years (mean (SD)) (*n* = 73,201)	7.4 (5.6)	6.8 (6.0)

**Table 2:** Visit Characteristics by Year.

Characteristic	April 2019 *n* = 77,560 (64.5%) *n* (%) or Mean (SD)	April 2020 *n* = 42,670 (35.5%) *n* (%) or Mean (SD)
Site (*n* = 120,230)		
1	18,198 (23.5%)	7969 (19.7%)
2	3807 (4.9%)	2136 (5.0%)
3	1978 (2.6%)	795 (1.9%)
4	53,577 (69.1%)	31,770 (74.5%)
Visit type (*n* = 119,627)		
Well visit	12,984 (16.9%)	5542 (13%)
Acute visit	64,020 (83.1%)	30,465 (71.5%)
Video visit	0 (0%)	4417 (10.4%)
Phone visit	0 (0%)	2199 (5.2%)
Visit diagnosis from ICD-10 codes (more than one or none could apply per visit)		
Well visit	13,220 (17.0%)	5908 (13.9%)
Asthma	2638 (3.4%)	943 (2.2%)
Obesity	2009 (2.6%)	505 (1.2%)
Attention deficit hyperactivity disorder	3773 (4.9%)	1767 (4.1%)
Depression	2193 (2.8%)	1089 (2.6%)
Anxiety	1899 (2.5%)	917 (2.2%)

**Table 3:** Characteristics Associated with Visit Attendance in 2020 ^a^.

Characteristic	Odds Ratio (95% Confidence Interval)
Male sex	1.01 (0.98, 1.03)
Race/ethnicity	
Non-Hispanic white	Ref
Non-Hispanic black	1.13 (1.09, 1.17) ***
Hispanic	1.08 (10.4, 1.12) ***
Other	1.04 (0.99, 1.09)
Insurance	
Medicaid	Ref
Other public	1.09 (1.03, 1.15) **
Private	1.04 (1.01, 1.07) **
None	1.48 (1.39, 1.58) ***
Age (years)	0.980 (0.978, 0.982) ***
Visit type	
Acute visit	Ref
Well visit	0.73 (0.71, 0.76) ***

^a^ Model adjusted for sex, race/ethnicity, insurance status, age, visit type, and site. Bold denotes *p* < 0.05. ** denotes *p* < 0.01, *** *p* < 0.001.

**Table 4:** Characteristics associated with visit attendance of specific visit types in 2020 ^a^.

Characteristic	Model 1: Well Visits Odds Ratio (95% CI)	Model 2: Asthma Odds Ratio (95% CI)	Model 3: Obesity Odds Ratio (95% CI)	Model ADHD Odds Ratio (95% CI)	Model 1: Depression Odds Ratio (95% CI)	Model 5: Anxiety Odds Ratio (95% CI)	Model 6: Telemed Visits Odds Ratio (95% CI)
Male sex	0.90 (0.85, 0.97) **	1.52 (1.33, 1.74) ***	1.25 (1.04, 1.50)	2.29 (2.27, 2.56) ***	0.62 (0.54, 0.71) ***	0.74 (0.68, 0.80) ***	1.07 (0.01, 1.13) *
Race/ethnicity							
Non-Hispanic white	Ref	Ref	Ref	Ref	Ref	Ref	
Non-Hispanic black	1.11 (1.01, 1.22) *	1.67 (1.40, 1.99) ***	1.13 (0.85, 1.52)	0.60 (0.72, 0.69) ***	0.46 (0.37, 0.57) ***	0.36 (0.31, 0.41) ***	0.86 (0.79, 0.93) ***
Hispanic	1.06 (0.96, 1.17)	0.88 (0.71, 1.09)	1.86 (1.43, 2.42) ***	0.32 (0.27, 0.39) ***	0.73 (0.60, 0.89) **	0.55 (0.48, 0.63) ***	0.94 (0.86, 1.02)
Other	1.28 (1.14, 1.42) ***	0.75 (0.57, 0.99) *	2.34 (1.73, 3.19) ***	0.51 (0.41, 0.64) ***	1.03 (0.82, 1.31)	0.63 (0.54, 0.75) ***	0.89 (0.81, 0.99) *
Insurance							
Medicaid	Ref	Ref	Ref	Ref	Ref	Ref	
Other public	0.76 (0.63, 0.91) **	0.89 (0.63, 1.25)	0.17 (0.06, 0.45) ***	1.07 (0.83, 1.36)	0.82 (0.58, 1.16)	0.91 (0.74, 1.10)	1.41 (1.24, 1.60) ***
Private	1.30 (1.20, 1.41) ***	0.78 (0.66, 0.92) **	0.47 (0.37, 0.60) ***	0.85 (0.75, 0.96) **	1.27 (1.09, 1.47) **	1.16 (1.06, 1.27) **	1.36 (1.28, 1.46) ***
None	1.83 (1.56, 2.14) ***	0.54 (0.30, 0.96) *	0.66 (0.33, 1.30)	0.39 (0.22, 0.68) **	0.95 (0.62, 1.45)	1.50 (1.20, 1.89) ***	0.75 (0.63, 0.90) **
Age (years)	0.75 (0.74, 0.76) ***	1.12 (1.10, 1.13) ***	1.15 (1.14, 1.17) ***	1.18 (1.17, 1.18) ***	1.23 (1.21, 1.25) ***	1.24 (1.23, 1.25) ***	1.07 (1.07, 1.08) ***

^a^ Model adjusted for sex, race/ethnicity, insurance status, age, and site. Bold denotes *p* < 0.05. * denotes *p* < 0.05, ** *p* < 0.01, *** *p* < 0.001.

## Data Availability

Restrictions apply to the availability of these data. Data were obtained from multiple institutions and are available from the authors with the permission of the institutions.
